# Current and Future Perspectives on the Management of *Helicobacter pylori*: A Narrative Review

**DOI:** 10.3390/antibiotics13060541

**Published:** 2024-06-10

**Authors:** Charlene Deane, Orlaith Kelly, Colm O’Morain

**Affiliations:** 1 Beacon Hospital Research Institute, D18 AK68 Dublin, Ireland; 2 Connolly Hospital, D15 X40D Dublin, Ireland; 3 Department of Medicine, Royal College of Surgeons Ireland, D02 YN77 Dublin, Ireland; 4 Department of Medicine, Trinity College Dublin, D02 PN40 Dublin, Ireland; 5 Tallaght University Hospital, D24 NR0A Dublin, Ireland

**Keywords:** *Helicobacter pylori*, treatment, screening, resistance, quadruple therapy

## Abstract

The prevalence of *Helicobacter pylori (H. pylori)*, a pathogen, has decreased globally in the last decade. To date, the management of *H. pylori* has focused on a reactive approach, whereby those diagnosed are treated with antimicrobials and acid suppression in combination. This review article provides an overview of the shift in the management of *H. pylori* from a reactive approach towards a proactive ‘screen and treat’ approach; the article reflects the current pharmacological landscape for *H. pylori* treatment by exploring similarities such as the first-line prescription of quadruple therapy in most countries and provides a summary table of the best practice guidance from Europe, Asia, and North America. It explores significant ongoing challenges in management, such as rising antimicrobial resistance rates, and explores a potential ‘work smart’ approach to antimicrobial susceptibility testing. We explore the role of registry databases in providing data on treatment efficacy and safety and how they can support a strategic approach to *H. pylori* treatment. We question if such a database’s availability, update, and regular audit should serve as a key quality indicator in a population screening programme. Despite a call for vaccination against *H. pylori* and decades of research, not many have made it to a phase-three clinical trial. We explore the challenges that have complicated the development of such a vaccine, such as the genetic diversity of *H. pylori*, immunotolerance, and limitations of mouse models in research; we reflect on how these challenges are contributing to a low likelihood of having a vaccine in the short–medium term. Lastly, it explores the heterogeneity in research on probiotics and their role as an adjunct in the management of *H. pylori*.

## 1. Introduction

*Helicobacter pylori (H. pylori)* was first discovered in 1982 by Warren and Marshall; before its discovery, the stomach was considered a sterile organ [1]. It is a Gram-negative, spiral-shaped, microaerophilic bacteria that colonises the gastric mucosa, with nearly all those chronically infected developing gastritis [2]. Twenty percent of those infected go on to develop ulceration, and one to three percent develop gastric cancer [3,4]. Specific patterns of gastritis are thought to be associated with different outcomes; for example, antral predominant gastritis is associated with duodenal ulceration and high acid secretion, and corpus gastritis is related to gastric cancer and low acid secretion [5]. Notable other common associations include iron-deficiency anaemia and immune thrombocytopenic purpura [6]. The majority of people infected are asymptomatic. However, it is still regarded as an infectious disease that requires treatment, regardless of the presence of symptoms or complications.

The prevalence of *H. pylori* is declining, previously reported to be as high as 50–55% worldwide; a recent meta-analysis of epidemiological studies supports a declining prevalence, with reported rates of 35% [7]. More recent population-based studies in Japan testing the prevalence in the adolescent cohort have even suggested rates as low as 3% [8]. Despite this falling prevalence, it remains a significant burden on the healthcare system and is unlikely to disappear spontaneously. Higher rates are reported in developing countries, Asian populations, and Eastern Europe, with lower rates in Western Europe. Why some people develop the infection and others do not is largely unknown. One theory is of an inherited genetic predisposition to the infection due to genetic variations in the host’s innate immune system. Toll-like receptor (TLR) proteins are important immune complexes and signal danger to the host cells. Genetic variations in these receptors and subsequent functional implications on recognising *H. pylori* have been researched. The effects on TLR-1, TLR-6, TLR-10, and the TLR-1 loci are hypothesised to play a role in the risk factors for infection, however the results remain inconclusive. Meta-analyses on the role of pro-inflammatory polymorphisms, such as the TNF-α-238G/A polymorphisms, have identified an increased risk of gastric cancer among Asians, but not among Caucasians, in individuals with *H. pylori* infection [9,10].

Population screening for *H. pylori* has been ongoing for decades in Asian countries; recently, a renewed interest has emerged in Europe, given the drive for a gastric cancer screening policy. While this drive is welcomed, it concurrently raises concern about the impact of widespread antibiotic use. Given a rise in antimicrobial resistance over the years an urgency already exists in the need for alternative solutions to conventional treatment for the management of *H. pylori*. Potential solutions include the development of new antimicrobials and pharmacological therapies, sensitivity testing prior to the use of existing antibiotics, and greater support in the development of a vaccine. We explore current screening recommendations, treatment guidelines, challenges and potential solutions in this article.

## 2. Methods

This narrative review article summarises developments in the indication for testing and the current pharmacological management of *H. pylori* using a combination of antimicrobials and acid-suppression agents. It explores the risk of antimicrobial resistance and the potential future role of probiotics and vaccination.

The references for this article were identified through PubMed, the Cochrane Database of Systematic Reviews, and the Cochrane Controlled Register of Trials with the search terms “gastric cancer”, “stomach cancer”, “Helicobacter pylori”, “vaccination”, “probiotics”, “resistance”, “antimicrobial”, “antibiotics”, and “screening” over the period from 1995 until April 2024.

## 3. Management: Developments on Who to Test for Infection

*H. pylori* is moving away from a ‘test and treat’ approach, whereby those with symptoms suggestive of infection are tested and treated, towards a ‘screen and treat’, whereby asymptomatic members of the population are screened for the infection and treated when present.

The primary driver in this change in approach is to reduce the incidence of gastric cancer. *H. pylori* has been classified as a type I carcinogen by the International Agency for Research on Cancer since 1994, and this status was reaffirmed in 2014 [11]. It currently stands as one of the 10 leading infectious causes of cancer worldwide by the National Cancer Institute [12].

*H. pylori* is the primary causative factor in 89% of gastric cancer cases. It has been well-established that, by interrupting the well-defined cancerous cascade known as the Correa cascade, whereby the mucosa progresses from superficial inflammation to gastritis, atrophy, intestinal metaplasia, dysplasia, and cancer, the incidence of gastric cancer can be reduced [3,13]. Given that this precursor state is easily tested for and treatable, it fulfils several of the Wilson–Junger criteria for screening and, therefore, is considered a potential primary preventative strategy against gastric cancer [14].

### 3.1. H. pylori Virulence Factors Influencing Gastric Cancer Risk

There are a number of modifiable and non-modifiable factors that can influence gastric cancer risk. These include smoking status, socioeconomic status, exposure to salt and nitrosamines, obesity, and alcohol. Family history is an important consideration. Those with a family history of gastric cancer are more likely to develop gastric cancer than the general population. Notably, 10% of gastric cancer cases diagnosed have a known family history; however, an identifiable genetic inherited mutation only accounts for 1–3% of cases [15]. The highest risks are in those with disruption in the CDH1 gene associated with hereditary diffuse gastric cancer syndrome, the STK11 gene associated with Peutz–Jeghers, and the SMAD 4 gene associated with juvenile polyposis [16].

The most significant risk factor is the presence of *H. pylori*. Why some people with this infection go on to develop gastric cancer and others do not is largely unknown. One hypothesis is that *H. pylori* virulence factors such as cytotoxin-associated gene A (CagA) and vacuolating cytotoxin A (VacA) strains increase the risk of gastric cancer [17]. VacA is a protein toxin that can be secreted by *H. pylori* and that has acute cytotoxic activity by inducing large intracellular vacuoles in the host. This ultimately results in cell death, autophagy, downregulation of immune response, and tolerance to *H. pylori*. Cytotoxin-associated gene A, a gene encoding the CagA protein in the bacterial genome, is thought to promote neoplastic transformation by manipulating intracellular signalling. The prevalence of CagA strains is higher in East Asia, with nearly all strains carrying the CagA-pathogenicity island (PAI), compared to only 30–40% of European strains; this is thought to contribute towards the high rate of gastric cancer identified in this region of the world [18,19].

### 3.2. Organisations Supporting the ‘Screen and Treat’ Approach

The International Agency for Research on Cancer (IARC), the Science Advisory Policy by European Academics (SAPEA), and the European Commission have called for a gastric cancer screening strategy as part of ‘Europe’s Beating Cancer Plan’ [20,21,22]. The Maastricht/Florence VI consensus guidelines also propose screening for *H. pylori* in countries that report an intermediate to high incidence of gastric cancer, this approach is also thought to be effective in lower-incidence countries also, however, less cost-effective [6]. Combined screening has also been proposed by Maastricht/Florence VI as a potential opportunity for gastric cancer screening. They suggest the combination of serological screening and upper gastrointestinal endoscopy at the time of scheduled colorectal cancer screening.

### 3.3. Transmission and Potential Preventative Strategies

Transmission of *H. pylori* most commonly occurs in childhood. It passes from human to human through an oral–oral route in developed countries or a faecal–oral route in developing countries. Transmission routes can be further subdivided into a vertical transmission route, i.e., from parent to child, or a horizontal route, i.e., infection from individuals outside the family or through environmental contamination [23,24]. The majority of *H. pylori* transmission is thought to occur before the age of 10 and to persist lifelong, while there is some evidence that a minority of children can clear infection sporadically [25]. Re-infection, distinct from the concept of recrudescence, is thought to be rare in developed countries, with an incidence of <1%. Recrudescence refers to the resurgence of the original strain of *H. pylori*, which is believed to be due to unsuccessful/incomplete eradication and only temporary repression of the bacteria [25].

Given the spread of *H. pylori* through the faecal–oral route, one cause of reduced transmission is thought to be driven by improved sanitation and hand-washing hygiene. The transmission from parent to child is also important to consider and it has been suggested that screening and treatment of parents prior to starting a family is another potential strategy to reduce transmission. Lastly, advertent or inadvertent intervention with antibiotics may have led to a reduction in transmission.

### 3.4. Screening Mechanisms

Testing for *H. pylori* can be performed via various methods: invasively through endoscopy with biopsy or non-invasively using serological antibodies, urea breath testing, or stool antigen testing. Urea breath testing is a sensitive (95–100%) and specific (95–100%) method of diagnosis, with the advantage of being non-invasive. Gastroscopy with biopsy has a sensitivity of 60–93%; however, it carries a risk of adverse events such as perforation, which, while rare, can occur. Monoclonal stool antigen testing has a reported sensitivity rate of >95%; however, it is affected by proton pump inhibitors, and compliance with the testing method can be difficult. Assays used in serology for *H. pylori* can vary in terms of sensitivity. A European review analysed 29 commercially available serological kits, which evaluated kits on 5 parameters: sensitivity, specificity, positive predictive value, negative predictive value, and accuracy, and found only 5 kits that achieved a score of >90% in all parameters [26]. The limitation of this method is that antibodies in serology can be present for months after infection, and, therefore, it cannot reliably distinguish between those with recent or active infection. Therefore, while it is of use in population studies, serology is generally only recommended for diagnosis in select clinical cases using assays that have been locally validated. In Europe, urea breath tests and CLO (campylobacter-like organism) testing at the time of gastroscopy are recorded as the two most common means of diagnosis in day-to-day practice [27].

### 3.5. Benefits and Challenges of a ‘Screen and Treat’ Approach

A proactive screening approach possesses many benefits, including (i) a reduction in the incidence of gastric cancer, (ii) a reduced attendance at primary care physicians for dyspepsia and other *H. pylori*-related symptoms, and (iii) reduced transmission to future generations as infected patients are a reservoir for transmission of the infection [28,29]. However, it is also essential to consider the challenges that this approach creates.

This approach raises questions as to (i) The best age to screen, given that infection typically occurs in childhood, and it is well-established that, the earlier people are screened, the greater the impact of reducing long-term sequelae. However, treatment of children is considered unnecessary given that they are unlikely to develop disease complications at this early stage [30,31]; (ii) The screening method must be sensitive, specific, and reproducible while considering resource requirements to fulfil screening. The choice of test may also positively or negatively impact the uptake rate, and a high uptake is necessary for the cost-effectiveness of a programme [32]; (iii) False positives and negatives can create false reassurance or unnecessary worry; (iv) There is concern over the potential impact of population screening and treatment on resistance rates and the gut microbiome. Data from the Matsu Island study related to this exist. They found no increase in antimicrobial resistance. Furthermore, a randomised control trial from Taiwan, which looked at the effect of second-line treatment on the microbiome, found that the diversity of the microbiota was largely restored between 2 months and 1 year after treatment completion [33]. We expect further research to emerge on this in the medium term as part of the GI-STAR study.

### 3.6. Recent and Ongoing Studies Supporting a ‘Screen and Treat’ Approach

Several randomised control trials have demonstrated the advantage of population screening in reducing gastric cancer risk. Ford et al. conducted a meta-analysis, encompassing seven randomised control trials involving healthy adults with *H. pylori* infection who received either treatment or a placebo [16]. The pooled analysis revealed a relative risk reduction in gastric cancer mortality for those receiving treatment (RR 0.54; 95% CI 0.40–0.72), with no heterogeneity among the studies. According to this meta-analysis, the calculated number needed to treat to prevent one case of gastric cancer was 72, and the number needed to treat to prevent one cancer-related death was 135. Numerous longitudinal observational studies also support the positive impact of population screening. The Matsu Island study, a notable prospective investigation, involved testing and treating 7000 adults over the age of 30 for *H. pylori*. The study reported a 53% reduction in gastric cancer incidence, a 23% reduction in mortality, and no increase in the antibiotic resistance rate of *H. pylori*. In a specific examination of a European population, Doorakkers et al. utilised the Swedish National database to assess the impact of *H. pylori* treatment on gastric cancer incidence, once again confirming the benefits of eradication in reducing mortality.

Ongoing studies in Asia include the Linqu County study in China and the HELPER (*Helicobacter pylori* Eradication for Gastric Cancer Prevention in the General Population) study from Korea [34]. In the HELPER study, *H. pylori*-positive patients were assigned to either an active or placebo arm. Participants were then followed for 10 years to determine the differences in the incidence of gastric adenocarcinoma post-eradication [35]. The Linqu County study is a large-scale prospective, randomised control study that aims to examine the impact of *H. pylori* screening and treatment on gastric cancer risk in a high-incidence country. Interim results have already identified a high prevalence of *H. pylori*, at 57.6%. Furthermore, risk factors that negatively affect eradication rates have been identified; these include male gender, smoking status, and high BMI.

In Europe, the Eurohelican and the TOGAS (Towards Gastric Cancer Screening Implementation in the European Union) studies are looking at an active ‘screen and treat’ approach in a young adult population to determine up-to-date prevalence, cost-effectiveness and feasibility of a screen and treat approach at the population level in countries with varying levels of prevalence of both *H. pylori* and gastric cancer. Meanwhile, in the UK, a longitudinal study, the *Helicobacter pylori* Screening Study (HPSS), will conclude in 2024 and aims to determine the potential impact of screening and treating *H. pylori* on gastric cancer risk over 10 years in a low-incidence country [36]. The GI-STAR study, based in a high-incidence country, Latvia, will examine the effect of *H. pylori* and serum pepsinogen screening on gastric cancer mortality and any adverse effects of doing so, including impact on the microbiome. It is due for completion in 2035 [37]. These studies hold promise in offering valuable insights into the effectiveness of *H. pylori* screening in adult populations in low–intermediate- and intermediate–high-risk European populations. They will also provide information on the potential long-term implications, cost-effectiveness, and adverse events of this approach. In doing so, these studies will guide European member states in implementing local policies.

In South America, Gallardo et al. in Chile are screening 14–18-year-olds for *H. pylori* to provide data on the feasibility and acceptability of this approach in a young adult cohort [38].

## 4. Management: Treatment

Current international guidance on the recommended treatment for *Helicobacter pylori*. Treatment aims to achieve successful eradication in >90% of cases. Recommended eradication regimens focus on combining acid suppression (proton pump inhibitor or potassium competitive acid blocker) with antibiotics. While most guidelines provide a framework for recommended regimens, it is important to consider the individual receiving the treatment. Factors such as allergy status, co-morbidities, resistance rates, and medication availability are all important considerations when deciding on treatment (Table 1). With rising resistance rates to clarithromycin, levofloxacin, and metronidazole, tailored therapy based on antimicrobial sensitivity testing (AST) is the target, and meta-analysis has indicated a superior eradication rate using this approach compared to quadruple therapy [39]. Unfortunately, access to AST is not always feasible, and guidelines that help physicians navigate treatment options are crucial.

### 4.1. First-Line Treatment

Current guidelines from Europe, Canada, the United States, and Korea support the use of bismuth-based quadruple therapy or concomitant quadruple therapy in areas where the resistance rate is unknown or >15%. The guidance on antibiotics for quadruple therapy differs (Table 2) and is generally guided by the antibiotics to which the patient has previously been exposed. PPI-based triple therapy is recommended in areas where clarithromycin resistance rates are <15%; however, this is becoming less common as resistance rates rise [6,43,44,45]. The WHO recognised the problem of clarithromycin-resistant infection in 2019 and listed it as a priority for antibiotic research and development [46].

The only guidelines that do not permit the role of quadruple therapy as first-line treatment are the Japanese guidelines. After reviewing its national data, Japan found insufficient evidence of the superiority of bismuth quadruple therapy over triple therapy. As a result, it does not recommend the first-line use of quadruple treatment except in a situation where no other listed options are available [47,48].

### 4.2. Second-Line Therapy

Fluoroquinolone-containing therapy is generally reserved as a second- or third-line therapy. Concerns around high resistance rates and its side effect profile, such as the risk of tendonitis and QT prolongation, restrict its use in practice. Despite this, it is an effective treatment. Real-world data from a European registry looked at over 5000 patients who received second-line therapy; this study confirmed that 14 days of levofloxacin–-bismuth therapy was one of 4 regimens with optimal effectiveness. The other regimens included 10-day single-capsule bismuth quadruple therapy, 14-day tetracycline, and 14-day quinolone triple therapy [49].

### 4.3. Rifabutin Therapy

Rifabutin triple therapy is generally reserved for treatment-resistant *H. pylori* infection. Concerns about its risk of bone marrow suppression and resistance to Mycobacterium are expressed. However, therapy tends to be relatively short, reducing the risk of exposure to side effects. A recent non-inferiority study of over 300 patients from China compared bismuth quadruple therapy with rifabutin triple therapy in those who had failed two prior lines of treatment for *H. pylori*. This study found an efficacy rate of >90% in the protocol and modified intention to treat groups in both arms. Furthermore, a lower rate of side effects and higher compliance was observed in those receiving the rifabutin-based therapy. It was noted that isolates with amoxicillin resistance were less likely to be successfully eradicated using rifabutin-based treatment, with a drop in eradication from 94.8% to 66.7%. It did not have the same effect on those in the bismuth group [50].

In Europe, the *H. pylori* EU registry (HpEUReg) examined the real-world data outcome in 500 cases treated using Rifabutin triple therapy. Eradication rates range from 68 to 80%. The HpReg found in practice that it was most commonly used as a second-line, third-line, or fourth-line therapy [51,52].

### 4.4. The Role of Proton Pump Inhibitors (PPIs) and Potassium Competitive Active Blockers (P-CABS) in Treatment

The critical role of acid suppression through proton pump inhibitors or P-CABs in improving the efficacy of *Helicobacter pylori* therapy is well-established in the literature [53,54]. PPIs and P-CABs both work by blocking the H+/K+ ATPase channel on the gastric parietal cells. However, PPIs bind covalently to proton pumps and have to be dosed around meals, whereas P-CABs bind ionically and bind both active and inactive proton pumps [55]. In doing so, both reduce gastric acid secretion, increase intragastric PH, and increase the bioavailability of antimicrobials. P-CABs hold an advantage over PPIs in that they have a quicker onset of action and a more extended treatment effect.

Genetic polymorphisms of CYP2C19 and their effect on PPI metabolism are thought to impact treatment success significantly. Polymorphisms are thought to occur in <4% of the European population but in more than 14% of the Asian population. There are three common polymorphisms: homozygous extensive metabolisers (which are more commonly found in a European population and result in quick rates of metabolism of PPIs), heterozygous, and poor-metaboliser genotypes, which tend to have slower metabolism of PPIs [56]. Slow metabolisers are believed to have the best effect on outcomes [57]. Specific PPIs are thought to be less affected by this than others, such as Rabeprazole. It is not thought that these polymorphisms have the same effect on P-CABs [58].

Given the superior performance of P-CABS, the question of whether these agents could be used more successfully in place of PPI treatment is raised. Japan recommends Vonaprazan or a PPI in their first-line treatment, and most other guidelines suggest considering P-CABs; however, access is less readily available in the United States and Europe.

A recent publication of a phase-three non-inferiority RCT conducted in a European and US population compared triple therapy co-administered with a PPI or a P-CAB. A higher eradication rate was found in those taking P-CAB triple therapy, both in clarithromycin-resistant strains and in the overall study population [59]. A non-inferiority study based in China compared bismuth quadruple therapy to dual therapy of amoxicillin four times daily with a P-CAB (Vonoprazan). This study supported the non-inferior efficacy of P-CAB with six hourly amoxicillin with eradication rates of >90%. It is worth noting that it did not achieve significance for those on amoxicillin dual therapy who only received amoxicillin twice daily [60]. Previous retrospective data from a European population did not find high-dose 8-hourly amoxicillin dual therapy to be an effective choice [61].

## 5. Management: Challenges

### 5.1. Antimicrobial Susceptibility Testing (AST)

Data on resistance rates in Europe in 2018 indicate resistance rates of 38.9% for metronidazole, 21.4% for clarithromycin, and 15.8% for levofloxacin [62]. In China, resistance rates are 63.8%, 28.9%, and 28%, respectively, and 29.5%,17.8%, and 37% in South Korea. Given the high resistance rates, attention has been given to the role of antibiotic susceptibility testing (AST) in treating *H. pylori*. AST is now incorporated into the guidelines, whereby treatment regimens can be guided based on the AST when and where available.

The two main ways to perform AST are culture-based and molecular methods, such as polymerase chain reaction (PCR) or next-generation sequencing [63]. Each technique comes with unique considerations. In the case of culture, a gastroscopy is required to obtain a biopsy for analysis; it is a costly, time-intensive process. Next-generation sequencing can be performed on both biopsy and stool samples. This process allows for the detection of resistance in a more timely manner. A recent article by Graham et al. reports that access to this is readily available in the United States through private laboratories [64].

### 5.2. Treatment Effectiveness: The Role of Registry Data

Registry databases are an essential component of treating *H. pylori*, as they can provide an overview of treatment resistance, eradication rates, and prescribing practices in areas. The European *Helicobacter pylori* registry is an example of such a database. Founded in 2013, it is an online database of patients diagnosed and treated for *H. Pylori* infection in Europe, with over 300 centres and more than 70,000 patients [65]. It has provided an overview of current prescribing practices and treatment efficacy across Europe and within participating countries. It has published over 18 studies evaluating the effectiveness of treatment, adverse event profiles, antibiotic resistance trends, and common mistakes in routine clinical practice [66].

Graham et al. support the strategic use of local databases to avoid the need for susceptibility testing on all patients. They suggest empiric therapy based on local susceptibility testing and AST in situations where empiric treatment fails to achieve eradication rates of >90% or where someone has failed first-line therapy [64]. Unfortunately, one of the limitations of such an approach is the ad hoc nature of how follow-up and eradication are performed from centre to centre and the lack of such a database in the United States [67].

## 6. Management: Future Direction for Treatment

Given the concern over antimicrobial resistance, there is a heightened focus on the development of new compounds for the treatment of *H. pylori*. Work on complementary strategies, such as the development of a vaccine and the role of probiotics, remains ongoing.

### 6.1. Probiotics and Prebiotics

Probiotics are living microorganisms that confer a health benefit on the host when administered in adequate amounts [68]. In the case of *H. pylori* infection, probiotics such as Lactobacillus reuteri can assist in a number of ways: they can (i) improve eradication by acting as a bacteriostatic agent, (ii) restore the gut microbiome post-treatment, and (iii) reduce treatment side effects. Furthermore, newer data suggest the use of engineered probiotics alone as treatment. That said, research on the use of probiotics is heterogeneous. There are a number of different types of probiotics which can be used in various combinations, and studies use various doses with different timing interval administration, in addition to variance within the host taking the probiotics [69]. As a result, outcomes from clinical studies have varied. Currently, the role of probiotics remains largely as that of an adjunctive therapy.

### 6.2. Vaccination

Given the success of HPV vaccination in reducing cervical cancer risk, it is plausible that vaccination would also be a feasible strategy for preventing the development of cancer through complications of *H. pylori* infection. Research into developing a vaccine against *H. pylori* has been ongoing for decades. Despite this, unfortunately, only a few have made it to the clinical trial stage, with only one making it to a phase III [70]. *H. pylori* possesses several unique and challenging strategies to help it survive hostile gastric environments and modify the host immune response to allow it to survive. As a result, no vaccine has yet succeeded in inducing long-term protection against *H. pylori* [71,72]. Additional barriers in the development of the vaccine include the limitations of the mouse model, the lack of financial interest in it compared to the likes of the COVID vaccine, the genetic diversity of *H. pylori* (with different strains of the same pathogen being seen in some studies), and the immunotolerance mechanism of t-regulatory cells that can allow tolerance to *H. pylori*. Furthermore, given that infection occurs in children, this vaccine needs to be suitable for children, have a high uptake rate, and have proof of long-term protection, which currently is lacking. While a lot of development has occurred in the field, the time to vaccine availability is still likely to be significant, as most studies are still in the early phase [71].

## 7. Future Direction

The management of *H. pylori* could potentially shift towards a ‘screen and treat’ technique that targets high-risk groups and areas. Vaccination is unlikely to be available in the short to medium term given the number of challenges listed above; however, there has been a significant body of work in the last 10 years, and the heightened focus on gastric cancer screening may further support the scientific effort to develop a vaccine. The role of probiotics is still largely that of an adjunctive therapy; however, research is ongoing, and it may provide a novel treatment strategy in the future. Newer antibiotics are urgently needed, as resistance rates pose a significant challenge worldwide. Until a time when newer strategies are available, the smart use of antimicrobial susceptibility testing to guide treatment will be important. The move towards a population-screening approach for *H. pylori* may provide an opportunity to create local registries, which could serve as key quality indicators in screening. Such registries would allow for regular audits of eradication confirmation for treatment success and identify areas whereby resistance is emerging to allow for targeted AST.

## 8. Conclusions

While the prevalence of *H. pylori* is decreasing, it is unlikely to disappear spontaneously. Infection with *H. pylori* is a significant clinical problem and should be treated, regardless of the presence or absence of symptoms. When deciding on treatment, it is important to consider access to AST and patient profiles. The most effective treatment documented locally should be prescribed, and strategies to optimise effectiveness, such as high-dose PPIs or P-CABs, should be implemented; registry databases are important components in implementing this. As the focus turns towards developing an effective gastric cancer screening strategy globally, the approach to testing for *H. pylori* may change in the coming years. Vaccination and engineered probiotics are potential solutions for the future; however, research is still in the early stages. In the interim, it would be important to consider the co-existent creation of local databases alongside any potential screening programme to permit a ‘work smart’ approach towards antimicrobial susceptibility testing and treatment success.

## Figures and Tables

**Table 1:** Antibiotics used in the treatment of *Helicobacter pylori* infection.

Antibiotic	Mechanism of Action (MOA)	Considerations
Amoxicillin	Competitively binds to penicillin-binding proteins, inhibiting transpeptidation, which results in the upregulation of autolytic enzymes. Causes the inability to repair and destruction of the cell wall.	Resistance rates are low (2–4%) Potential use as monotherapy with P-CAB. Can achieve an MIC for >24 h if dosed QDS.
Clarithromycin	Penetrates bacterial cell walls, binds to subunit 50 s of the bacterial ribosome, resulting in an inhibition of protein synthesis in bacteria.	High resistance rates (17.8–38.5%) QT prolongation agent
Metronidazole	Inhibits protein synthesis by interacting with DNA; causes a loss of helical structure, strand breakage, and cell death.	High resistance rates (29–63%) Dose needs to be high to optimize response (i.e., >1500 mg/day × 14 days)
Tetracycline	Reversibly binds to the bacterial ribosomal 30S subunit, inhibiting the elongation phase of RNA synthesis.	Not suitable for children due to effect on bone growth Risk of hepatotoxicity
Bismuth	Bismuth salts contain bactericidal and antimicrobial activity and prevent bacteria from binding and growing on the mucosal cells of the stomach.	Bacteriostatic effect, not altered by resistance Synergy with antibiotics, improving eradication rates in those with resistant infection Lack of availability of single capsule in certain countries
Levofloxacin	Directly inhibits bacterial DNA synthesis promoting the breakage of DNA strands by inhibiting DNA-gyrase, which inhibits the relaxation of supercoiled DNA.	High resistance rates in certain regions (15–37%) Use with caution in elderly and those with co-morbidities Risk of tendonitis QT prolongation Risk of heart valve regurgitation
Rifabutin	Inhibits RNA polymerase in bacteria, leading to a suppression of RNA synthesis and cell death.	Risk of bone marrow suppression Concern about inducing resistance to Mycobacterium tuberculosis Recent evidence proving non-inferiority to quadruple therapy

References: [40,41,42].

**Table 2:** Summary of treatment guidelines.

Guidelines	1st-Line Treatment	2nd-Line Treatment	Rescue Treatment i.e., 3rd- or 4th-Line Treatment
Management of *H. pylori* infection: the Maastricht VI/Florence consensus report (2022)	CLT resistance > 15% BQT × 14 days * Non-BQT × 14 days # CLT resistance < 15% BQT × 14 days * CLT triple therapy × 14 days	LFX quadruple × 14 days BQT × 14 days * PPI or P-CAB + AMO	RIF triple therapy
Evidence-Based Guidelines for the Treatment of *H. pylori* Infection in Korea (2020)	Resistance > 15% Triple therapy × 14 days Quadruple sequential therapy × 10 days Quadruple concomitant therapy × 10 days CLT-based triple therapy × 7 days permitted in sensitive strains in PCR testing	BQT × 14 days	LVX triple therapy
Guidelines in Japan (2019)	CLT triple therapy with P-CAB or PPI × 7 days	MET triple therapy with P-CAB or PPI × 7 days	SIT triple therapy High dose PPI dual therapy
Fifth Chinese National Consensus Report on the management of *H. pylori* infection (2018)	No specific ‘1st or 2nd line treatment’. To be guided by resistance and chose the treatment most likely to achieve eradication. In general, recommend: BQT × 14 days		Rescue regimens guided by previous regimens
	Amoxicillin 1 gm BD + 1 of CLT 500 mg BD MET 400 mg TDS/QDS LFX 500 mg QDS/200 mg BD FUR 100 mg BD/TDS TET 500 mg TDS/QDS OR TET with 1 of MET 400 mg TDS FUR 100 mg BD		
The Toronto Consensus for the Treatment of *H. pylori* Infection in Adults (2016)	Resistance > 15% BQT × 14 days * Concomitant non-BQT # × 14 days CLT resistance < 15% Triple therapy~ × 14 days	BQT * Non-BQT # (Antibiotics used guided by what was already received)	RIF triple therapy
ACG Clinical Guideline: Treatment of *H. pylori* Infection (2016)	Resistance > 15% BQT * × 10–14 days LFX triple × 14 days LFX sequential × 14 days CLT resistance < 15% BQT * × 10–14 days Triple therapy~ × 14 days	BQT RIF triple therapy LFX triple therapy High dose metro triple High dose dual	

BQT = Bismuth quadruple therapy, LFX = Levofloxacin, MET = Metronidazole, TET = Tetracycline, CLT = Clarithromycin, AMO = Amoxicillin, RIF = Rifabutin, SIT = Sitofloxacin, BD = twice daily, TDS = three times daily, QDS = four times daily, * Bismuth quadruple (BQT) = Bismuth, Tetracycline, Metronidazole, Proton pump inhibitor (PPI). # Non-bismuth (non-BQT) concomitant = Clarithromycin, Metronidazole, Amoxicillin, PPI. Levofloxacin quadruple = Levofloxacin, Amoxicillin, Bismuth, PPI. Rifabutin triple = Rifabutin, Amoxicillin, PPI. Triple therapy = PPI, Clarithromycin, plus one of amoxicillin or metronidazole.

## Abbreviations

*Helicobacter pylori (H. pylori)*, Immunoglobulin G (IgG), Campylobacter Like Organism (CLO), Toll-like receptor (TLR), Cytotoxin-associated gene A (CagA), Vacuolating cytotoxin A (VacA), Serine/threonine kinase 11 (STK11), Towards Gastric Cancer Screening Implementation in the European Union (TOGAS), International Agency for Research on Cancer (IARC), The Science Advisory Policy by European Academics (SAPEA), antimicrobial susceptibility testing (AST), *Helicobacter Pylori* Eradication for Gastric Cancer Prevention in the General Population (HELPER), Bismuth quadruple therapy (BQT), Levofloxacin (LFX), Metronidazole (MET), Tetracycline (TET), Clarithromycin (CLT), Amoxicillin (AMO), Rifabutin (RIF), Sitofloxacin (SIT), Proton Pump Inhibitors (PPIs), Potassium Competitive Active Blockers (P-CABS).
